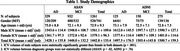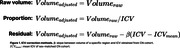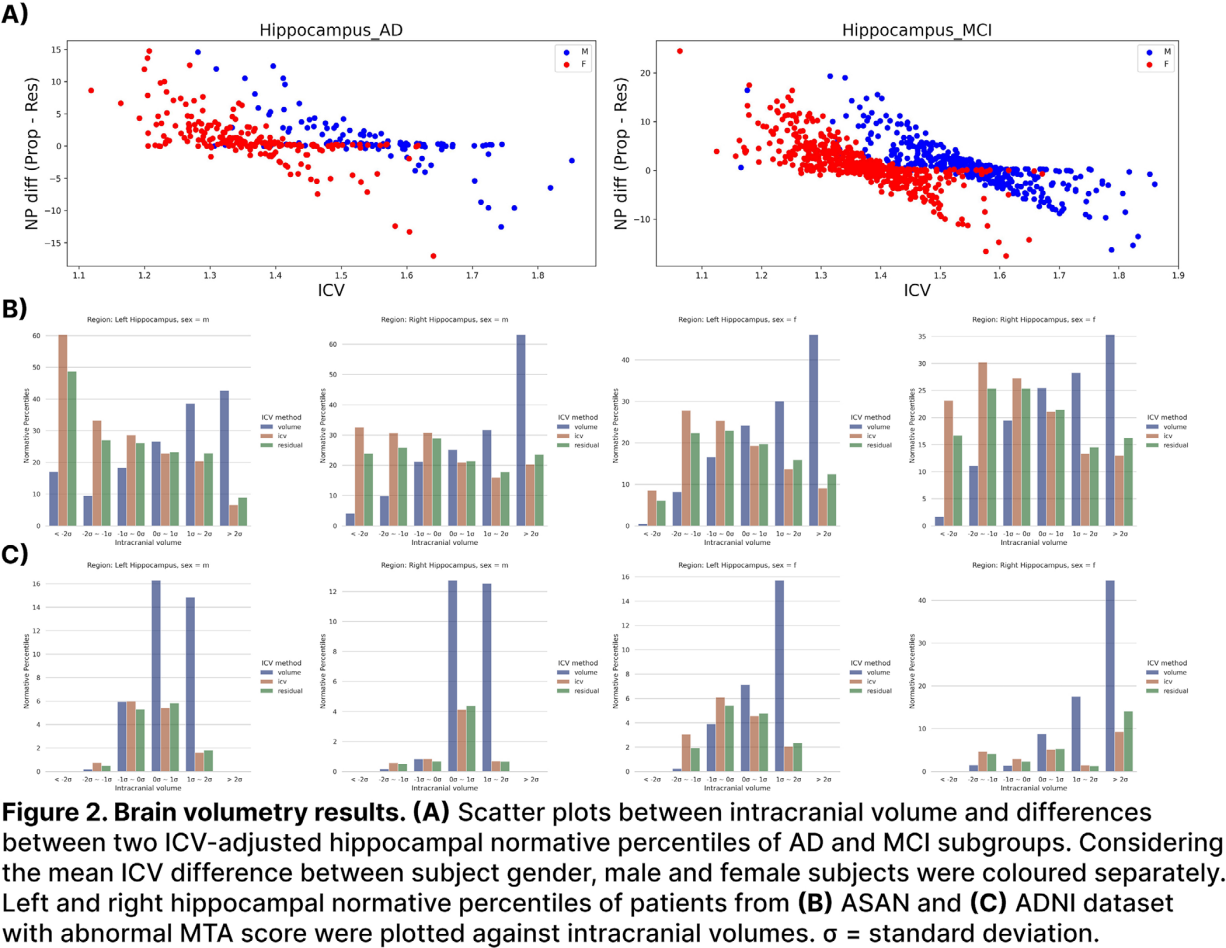# Comparison of intracranial volume adjustment methods to evaluate brain atrophy severity in AD continuum

**DOI:** 10.1002/alz.093715

**Published:** 2025-01-09

**Authors:** Wooseok Jung, Chong Hyun Suh, Seung Hyun Lee, Jinyoung Kim, Dong‐Hee Kim, Hyeonwoo Cho, Yeha Lee, Sang Joon Kim

**Affiliations:** ^1^ VUNO Inc., Seoul Korea, Republic of (South); ^2^ Asan Medical Center, University of Ulsan College of Medicine, Seoul Korea, Republic of (South)

## Abstract

**Background:**

Normative percentile (NP) quantifies brain atrophy by comparing regional brain volumes of a subject against age and sex‐matched cognitively normal populations. Accurate intracranial volume (ICV) adjustment is vital in NP quantification to minimize the effect of an individual’s head size. However, which intracranial volume adjustment method yields reliable normative percentiles remains unclear. This study explores the differences between ICV adjustment methods to compute NPs and their accuracy in atrophy quantification.

**Methods:**

We sampled MRIs of 1261 subjects consecutively visited the memory clinic (932 MCI; 329 AD dementia) for memory concern and 275 from ADNI (150 MCI; 125 AD). We utilized the AI‐based ICV segmentation tool implemented in VUNO‐Med DeepBrain to measure ICV. We compared three ICV adjustment methods for the NP computation: raw volume, proportion, and residual approaches (Figure 2). To evaluate the reliability of NP, we gauged the correlation between left and right hippocampal NPs and their medial temporal lobe atrophy (MTA) scores manually annotated by two neuroradiologists with consensus. The cut‐off of the MTA score of each hemisphere is set to 2 for subjects with age < 75 and 3 otherwise.

**Results:**

All ICV adjustment methods effectively reduced correlation with ICV (correlations: raw volume = 0.32±0.10, proportion = ‐0.06±0.09, residual = ‐0.07±0.08), but there was no statistically significant difference between the correlations of the proportion and residual methods to ICV. Also, the proportion method retrieves larger hippocampal NP from patients with smaller ICV than the residual method in all disease stage groups. Plotting the mean normative percentiles against ICV volumes suggests that the raw volume method generates more reliable NPs to detect abnormalities in patients with smaller ICV, but using the proportion method was more effective in those with large ICV in both ADNI and ASAN datasets.

**Conclusion:**

Different ICV adjustment methods generate distinct normative percentiles. Despite their effective head‐size correction, applying ICV adjustment was only more effective in subjects with larger ICV than average. Further research is required to confirm if this result applies to other brain regions.